# Pulmonary surfactant is indispensable in order to simulate the in vivo situation

**DOI:** 10.1186/1743-8977-10-6

**Published:** 2013-03-25

**Authors:** Carsten Schleh, Wolfgang G Kreyling, Claus-Michael Lehr

**Affiliations:** 1 Department of in vivo Pharmacology/Toxicology, BSL BIOSERVICE Scientific Laboratories GmbH, Behringstr, 6/8, Planegg/Munich 82152, Germany; 2 Institute of Epidemiology II, Helmholtz Center Munich – German Research Center for Environmental Health, D-85764, Neuherberg/Munich, Germany; 3 Helmholtz institut for Pharmaceutical Research Saarland (HIPS), Helmholtz Center for Infection Research (HZI), Saarland University, 66123, Saarbrücken, Germany

**Keywords:** Nanoparticle, Lung, Pulmonary surfactant, Coating

## Abstract

The article of Gasser et al. [Part Fibre Toxicol. 24; 9:17, 2012] describes the interaction of carbon nanotubes with cells within a complex cell culture model. Besides various toxicity parameters, the influence of coating with pulmonary surfactant was investigated. Pulmonary surfactant covers the entire alveolar region with the main function of decreasing the surface tension in the alveoli to prevent alveolar collapse. Although each inhaled nanoparticle, reaching the alveoli, will come into contact with pulmonary surfactant which will probably lead to a surfactant coating, pulmonary surfactant components are not commonly integrated in *in vitro* systems. Gasser and co-workers have shown that this surfactant coating is able to influence the further interaction with cellular systems. Hence, each scientist, working with *in vitro* systems and nanoparticles, should think of integrating pulmonary surfactant structures in order to harmonize the *in vitro* systems with the in vivo situation. In the present commentary we discuss the most important points of the manuscript of Gasser et al. and discuss where the usage of pulmonary surfactant can be further optimized.

## Background

The article of Gasser and co-workers [1] describes the interaction of carbon nanotubes (CNTs) with an *in vitro* cell culture model – uptake of CNTs as well as their ability to cause oxidative stress, cytokine/chemokine release and induction of apoptosis were evaluated. In some experiments, CNTs were pre-coated with the porcine pulmonary surfactant preparation Curosurf® before incubation with the *in vitro* system.

## Main text

Curosurf® is a commonly used pulmonary surfactant preparation [2,3]. The influence of this surfactant-coating on the interaction with an *in vitro* cell system was recently investigated by Gasser et al. [1]. Although the described experimental approach is very simple and easy to perform, the obtained results are very important and bring more light into the darkness of what are the crucial factors in nanoparticle toxicity. Furthermore, these experiments display an important step in making *in vitro* systems more similar to the in vivo gold standard.

Pulmonary surfactant consists of phospholipids and the four surfactant proteins (SP)-A, -B, –C, and -D. It covers the entire alveolar region with the main function of decreasing the surface tension in the alveoli to prevent alveolar collapse. Furthermore, surfactant components participate in the immune defence. Each individual inhaled nanoparticle, which reaches the alveolar region, will always impinge on pulmonary surfactant. Pulmonary surfactant is thereby the first contact of the human body with the inhaled nanoparticle. This first contact leads to a coating of the nanoparticle with surfactant lipids [4,5] and also surfactant proteins [6-10]. No uncoated nanoparticle will ever reach lung cells. Since nanoparticles interact by means of their surface with their environment the respective coating will consequently influence the interaction of the inhaled particles with lung cells. Within the present manuscript the group of Barbara Rothen-Rutishauser has clearly shown that surfactant-coating of CNT is able to change their toxic behaviour. Hence, the investigation of the influence of this surfactant coating is not only consequent but an absolute must in order to understand the mechanism of nanoparticle toxicity. This altered toxic behaviour may be due to a changed direct interaction with the cells or due to an affected uptake of NP into individual cells [11,12].

This knowledge will increase our competence to engineer safe nanoparticles which serve but do not harm humans and should be expanded to other research groups performing *in vitro* testing. The development of viable alternatives to in vivo tests is one of the five grand challenges in nanotechnology as postulated by Maynard et al. [13] to evaluate the toxicity of engineered nanomaterials during the next 15 years. Although several *in vitro* systems exist and although there is no doubt that most of these systems lead to a good first insight into the toxicity or even to a better mechanistic understanding of the toxicity there is also no doubt that these systems have to be optimized in the future. A valid alternative to the gold standard in toxicity testing – the in vivo system – has to be created. Such in vivo-near *in vitro* systems will lead to:

– decreased costs compared to expensive in vivo studies,

– results within a relatively short time,

– and also to an optimized animal welfare due to minimising animal testing.

Besides the simple *in vitro* monocultures complex co-culture systems – like the model in the present study of Gasser et al. – can be used. However, as we know from the present manuscript a relevant coating of the nanoparticles should be used in order to optimize *in vitro* testing. Especially when focusing on the lung a surfactant coating should always be integrated in the experimental design. Furthermore, more details should be optimized in order to reach a fine tuning of the experimental approach. In the experiments of the present manuscript, the CNTs where first coated with surfactant and then pipetted into the medium of the submersed cell culture. This simulates not directly the in vivo situation where particles impinge on the surfactant layer and afterwards are pushed into the aqueous hypophase by surface forces [4,14]. In this hypophase nanoparticles come into contact with various surfactant- and also other proteins which may interact with the surfactant phospholipid layer at the outer particle surface. In the present submerse cell culture model just components from the cell culture medium exist which may interact with the CNTs and its phospholipid coating. The biggest source for proteins in cell culture media is added serum (e.g. fetal calf serum), which was not incorporated in the study of Gasser et. al. However, in the RPMI1640 medium, which was used in the present study, also peptides like gluthatione are present which of course may interact with the particles or fibres. This scenario does not resemble the in vivo situation. Hence, air liquid interface models with a thin surfactant layer at the top should be the preferred choice when working with *in vitro* models to investigate the toxic effects of nanoparticles. Furthermore, although Curosurf® is a suitable model for pulmonary surfactant, it is not ideal and a better alternative should be found. Curosurf® mainly contains lipids and the surfactant proteins B and C. The surfactant proteins -A and-D are hardly found. Since it is known, that SP-A and SP-D bind to nanoparticles and may influence the toxicity, uptake into cells as well as biodistribution of nanoparticles [8,15] a surfactant preparation containing phospholipids as well as all four surfactant proteins should be found. However, due to the general method of surfactant isolation a balanced presence of the proteins is rather complicated since SP-A and –D are hydrophilic while SP- C and –B are lipophilic. Further surfactant preparations are commercially available and the most common are Alveofact, Infasurf, and Survanta. As Curosurf, none of the other commercial surfactants contain SP-A and SP-D since all are lipid extracts. Furthermore, they differ regarding their amount of SP-B and SP-C, the phospholipid composition as well as the source (Curosurf is porcine; Alveofact, Infasurf, and Survanta are bovine). Due to the different concentrations of proteins and phospholipids a differed interaction with the particles and subsequently a different outcome of the toxicological response can be expected. An even more in vivo like situation may be given, if not a commercially available surfactant preparation is used but lung lavage fluid in which also SP-D and SP-A among all other lung lining fluid proteins are available.

Since the coating of proteins on the particles surface is not a static but a dynamic process, depending on affinities, quantities and of course kind of available proteins, the usage of different surfactant preparations or lavage fluids will lead to different binding dynamics on the surface of the particle. Furthermore, also lipids compete for the space at the particles surface. Additionally, proteins can bind directly on lipids bound on the surface. Hence, since the bound proteins or lipids will come into contact with cellular structures, these dynamics influence the toxic response and the best surfactant preparation or lavage fluid has to be investigated in order to mimic the in vivo situation in an *in vitro* model.

Besides the direct interaction with lung cells after inhalation, surfactant coating is also able to determine the toxic effects in secondary target organs like the liver:

1) The initial coating in the surfactant layer will influence the subsequent interactions with cell and organ membranes leading to dynamic exchange of the particle coating and hence continues to affect the biodistributional fate of the particle in the various organs and tissues.

2) The number of particles, reaching secondary target organs is influenced by coating. It is clearly known, that nanoparticles can cross the air-blood barrier to reach nearly each organ [16] and that coating of nanoparticles influences this biodistribution. Hence, since surfactant is at least the initial coating of the nanoparticles, it will determine the toxic response in secondary target organs. Although we know that proteins and lipids at the surface of the nanoparticle are exchanged by time, the initial coating has a significant influence on this process [17]. This should be considered for *in vitro* experiments with e.g. liver cells.

At the end it is important to note, that not only surfactant is able to modify the particles mode of harm in cellular structure but also particles are able to affect the function of pulmonary surfactant. This is especially known for biophysical surfactant function when particles increased the surface tension at the air-liquid interface of pulmonary surfactant [18-20].

## Discussion and conclusion

The following take home messages can be concluded:

1) Inhaled nanoparticles which deposit in the alveoli will be coated with pulmonary surfactant

2) Pulmonary surfactant coating is able to influence the toxic response in the lung and also in secondary target organs

3) An suitable experimental step of pulmonary surfactant coating should be integrated in optimised *in vitro* systems in order to simulate the in vivo situation

## Abbreviations

CNTs: Carbon nanotubes; SP: Surfactant proteins.

## Competing interests

The authors have declared that no competing interests exist.

## Authors’ contributions

CS drafted the manuscript, WGK and CML revised the manuscript critically for important intellectual content. All authors read and approved the final manuscript.
